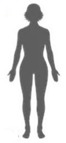# Microalgal Carotenoids: A Review of Production, Current Markets, Regulations, and Future Direction

**DOI:** 10.3390/md17110640

**Published:** 2019-11-13

**Authors:** Lucie Novoveská, Michael E. Ross, Michele S. Stanley, Rémi Pradelles, Virginie Wasiolek, Jean-François Sassi

**Affiliations:** 1 Scottish Association for Marine Science (SAMS), Scottish Marine Institute, Oban PA37 1QA, UK; Michael.Ross@sams.ac.uk (M.E.R.); Michele.Stanley@sams.ac.uk (M.S.S.); 2 Microphyt, 713 Route de Mudaison, 34670 Baillargues, France; remi.pradelles@microphyt.eu (R.P.); Virginie.Wasiolek@microphyt.eu (V.W.); 3 Commissariat à l’énergie atomique et aux énergies alternatives (CEA), Centre de Cadarache St Paul Lez, 13108 Durance, France; Jean-Francois.SASSI@cea.fr

**Keywords:** microalgae, carotenoids, mass production of microalgae, regulations, legislation, antioxidants, feedstock

## Abstract

Microalgae produce a variety of compounds that are beneficial to human and animal health. Among these compounds are carotenoids, which are microalgal pigments with unique antioxidant and coloring properties. The objective of this review is to evaluate the potential of using microalgae as a commercial feedstock for carotenoid production. While microalgae can produce some of the highest concentrations of carotenoids (especially astaxanthin) in living organisms, there are challenges associated with the mass production of microalgae and downstream processing of carotenoids. This review discusses the synthesis of carotenoids within microalgae, their physiological role, large-scale cultivation of microalgae, up- and down-stream processing, commercial applications, natural versus synthetic carotenoids, and opportunities and challenges facing the carotenoid markets. We emphasize legal aspects and regulatory challenges associated with the commercial production of microalgae-based carotenoids for food/feed, nutraceutical and cosmetic industry in Europe, the USA, the People’s Republic of China, and Japan. This review provides tools and a broad overview of the regulatory processes of carotenoid production from microalgae and other novel feedstocks.

## 1. Unlocking the Potential of Microalgae

Microalgae are a diverse group of microscopic plants. They are found in all aquatic environments across the entire planet [1]. Common ancestors of microalgae (DNA based microbes) lived in aquatic environment > 3.5 billion years ago and during that time, microalgae evolved and diversified [2]. Today, there are hundreds of thousands of different species; some adapted to thrive in the most extreme environments ranging from acidic mine waters and desert crusts to life in snow [3,4]. Microalgae serve at the bottom of aquatic food chains, fix carbon and significantly contribute to oxygen production on earth [5]. In addition to their fundamental ecological role, microalgae are being exploited commercially. Microalgae have been documented to produce a variety of beneficial compounds such as anti-cancer, anti-inflammatory, and anti-fungal compounds, antioxidants, vitamins, minerals, omega-3 fatty acids, pigments, and many others [6]. However, only a fraction of microalgal species have been scientifically described (~44,000) and an even smaller fraction (~3000) is being kept in culture collections for research [7]. The number of microalgal species that are currently used in commercial application is less than 40 strains. Given that thousands of microalgal species remain undiscovered, undocumented and unutilized, their biological diversity and potential for economic exploitation of microalgae is very high [8]. 

## 2. Carotenoid Classification and History

Carotenoids are pigments derived from tetraterpenes, which are compounds consisting of 8 isoprene (C5) units comprising a 40-carbon polyene structure. Terpenes are the largest family of natural organic products and metabolites. There are over 1100 naturally occurring carotenoids produced by > 600 source organisms, including plants, algae, fungi, bacteria, and archaea [9]. Carotenoids can be categorized into two groups: oxygen-containing xanthophylls (e.g., astaxanthin and zeaxanthin) and carotenes which are pure hydrocarbons with no oxygen (β-carotene and lycopene). Carotenoids are fat-soluble, absorb light within a wavelength of 400–550 nm and are the source of yellow, orange and red colour of many fruits and vegetables. They range from colourless to deep-red, with their colour dependent upon the number of conjugated double bonds [10]. Carotenoids cannot be synthesized by humans; we must obtain a sufficient amount from our diet. 

**Figure 1:**
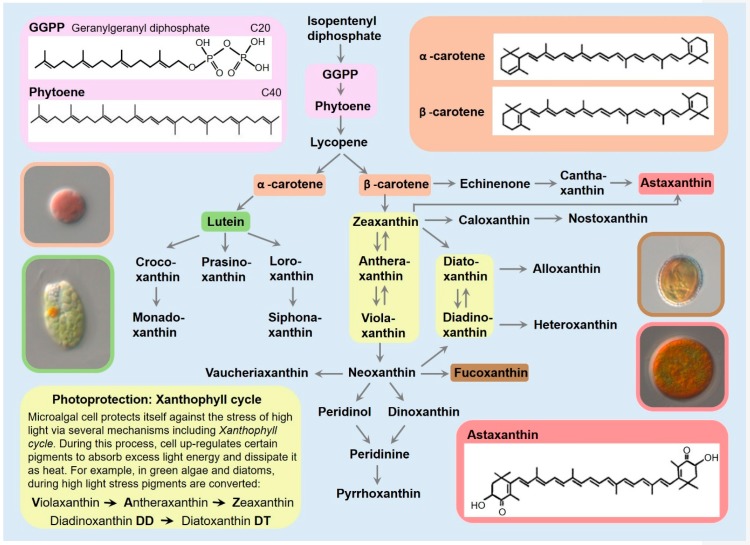
Carotenogenesis (selected pathways and molecular structures) (adapted from [14]). *Porphyridium* sp. (orange border) which contains β-carotene, *Tetraselmis* sp. (green border) which contains lutein, *Pleurochrysis* (brown border) which contains fucoxanthin and *Haemotococcus* sp. (pink border) which contains astaxanthin.

Carotenoid nomenclature is classified by their two group endings. There are seven different group ends in carotenes and each group end is denoted by a different Greek letter (β, γ, ε, χ, κ, ϕ, ψ). Carotene names have two Greek letters as prefixes based on these group ends. For example, β, ε carotene (common name α- carotene) contains one β ring and one ε ring. β, β carotene (β-carotene) contains two β ends and 3,3’-dihydroxy-β,β-carotene-4,4’-dione (astaxanthin) contains two β rings as well (Figure 1).

The first carotenoid and its benefits were discovered in 1831, when German scientist W. H. Wackenroder discovered carotene in carrot juice. In the early 20th century, Harry Steenbock and his colleagues suggested a relationship between vitamin A activity and carotenoid pigmentation [11]. However, it was not until 1931 that the structure of β-carotene was described by Swiss scientist Paul Karrer who received Nobel Prize in Chemistry for his discovery. The number of described carotenoids has increased linearly since that time at average of 15 a year [9]. 

## 3. Carotenoid Synthesis inside Microalgal Cells

Microalgae have the capacity to produce carotenoids and all known xanthophylls found in terrestrial plants (e.g., zeaxanthin, lutein, antheraxanthin). In addition, they can synthesize a variety of additional pigments that are specific to only algae, cyanobacteria, and several species of yeast (e.g., astaxanthin, fucoxanthin, diatoxanthin, diadinoxanthin) [12,13]. Some carotenoids are specific to certain algal classes, which means these pigments can serve as chemotaxonomic markers. For example, fucoxanthin is found in brown algae and diatoms, peridinin in dinoflagellates, and alloxanthin in cryptophytes [14]. Carotenoids may be divided into two categories: (A) primary carotenoids that are components of photosynthetic apparatus (both structural and functional) and therefore, are essential for survival and (B) secondary carotenoids that are produced via *carotenogenesis* only when cells are exposed to specific environmental conditions, such as high light, osmotic shock, etc. [12]. While primary carotenoids are associated with membranes, secondary carotenoids are located in lipid vesicles in the stroma of a plastid or the cytosol. However, some carotenoids synthesized in the chloroplasts can be exported into the cytoplasm and are consequently found anywhere in the cell [15].

Carotenoids perform important functions during photosynthesis, including light harvesting and photoprotection. As accessory pigments, in low light, carotenoids harvest available light and pass the energy to the chlorophyll molecules. During exposure to high light, the opposite occurs, carotenoids accept excess energy from chlorophyll and dissipate it—hence protecting chlorophyll from photodamage. Carotenoids protect microalgal cells by quenching triplet chlorophyll states and physically stabilizing the membrane. Carotenoids are able to neutralize free radicals and reactive oxygen species. It is this antioxidant ability that protects microalgal cells and gives them potential commercial applications for human consumption [16] (Table 1).

Microalgae are a suitable feedstock for the commercial production of carotenoids. The production of astaxanthin from microalgae is profitable and already established on a global scale because microalgae dominate natural astaxanthin production per unit dry weight (DW). For example, microalga *Haematococcus pluvialis* can produce astaxanthin at > 4% per DW [17], which is favourable when compared to the bacterium *Paracoccus carotinifaciens* (2.2% DW) [18], the yeast *Phaffia rhodozyma* (<0.5% DW) of (3R,3’R)-astaxanthin [19,20] and shrimp/crab shells (<0.025% DW) [21]. The commercial production of beta-carotene is increasing as well; meanwhile, other less established pigments (e.g., lutein, zeaxanthin, fucoxanthin) are also gaining momentum. Marigold flowers are the current primary commercial source of lutein; however, it has been demonstrated that microalgae can contain three to six times as much per unit mass. In comparison to terrestrial plant production, algae need significantly less water and land but may require greater energy and nutrient inputs [22].

## 4. Carotenogenesis

Carotenoid synthesis is complex, involving dozens of genes and enzymes, the description of which is beyond the scope of this review. Instead, we provide a summarized overview (Figure 1); for a fuller account, please see [23]. 

Isopentenyl diphosphate (IPP) contains five carbon atoms (C5); it is the isoprenoid precursor and the building block of all terpenes and carotenoids. Geranylgeranyl diphosphate (GGPP), containing 20 carbons atoms (C20) is synthesized from four IPP molecules. During condensation of the two C20 compounds (GGPP), the first carotene precursor, phytoene (C40), is formed by phytoene synthase. Phytoene is converted to lycopene during four desaturation steps. Lycopene is then cyclized on both ends to form β-carotene (Section 2). Identical hydroxylation of both β-carotene rings yields zeaxanthin that can be epoxidated once to form antheraxanthin or twice to form violaxanthin. Neoxanthin and other pigments are derived from violaxanthin, zeaxanthin or β-carotene [12,14,24]. There are two main pathways for synthesis of astaxanthin in microalgae. In one pathway, the enzyme carotenoid hydroxylase converts canthaxanthin into astaxanthin and in the second pathway, the enzyme β-carotene ketolase forms astaxanthin from the zeaxanthin branch, during which multiple intermediate molecules are involved (Figure 1) [25].

Green algae contain α-carotene and its derivatives. Similarly to β-carotene, α-carotene is formed from lycopene. The hydroxylation of the β- and ε-rings of α-carotene forms lutein, from which other carotenoids are derived (Figure 1) [12,14].

The zeaxanthin- antheraxanthin- violaxanthin pathway (VAZ pathway) as well as the diadinoxanthin- diatoxanthin pathway (DD-DT pathway) (Figure 1) are reversible and serve as cell energy dissipation systems during high light. This photoprotection mechanism is called the xanthophyll cycle. These pigments serve as additional buffers and are upregulated in conditions of high light to avoid photodamage [26].

## 5. Commercial Applications

The global carotenoids market was valued at $1.24 billion in 2016 and is projected to reach $1.53 billion by 2021 [27]. For this forecasted period, the carotenoids market is led by astaxanthin followed by β-carotene and lutein. Among all existing carotenoids, β -carotene, lycopene, astaxanthin, zeaxanthin, and lutein are considered as the most relevant due to their established applications. However, fucoxanthin, canthaxanthin and even the colourless carotenoid precursor phytoene are entering the market with potential scope for economic significance [28]. 

Practical applications of carotenoids can be found in food, feed, cosmetics and nutraceutical industries. Carotenoids have been traditionally used in the food and feed industries because of their colour and nutritional properties. Carotenoids are considered as a safe natural dye and as such, are added to a variety of products to enhance their colour (e.g., juices, yoghurts, confectionary). The animal feed sector was the highest consumer of carotenoids in 2014 with 47% market share. This segment is projected to grow from $562 million in 2014 to $665 million in 2019 at a 3.4% compound annual growth rate (CAGR) [29]. In the poultry diet, carotenoids contribute to the desirable colour of the egg yolk [30,31]. Similarly, in aquaculture, carotenoids have been supplemented to the diet of aquatic organisms to promote the desirable pink/red colour of crustaceans, salmonids and other farmed fish [32]. Aquaculture is the fastest growing animal food production sector worldwide [33], for example, Scottish aquaculture, which is dominated by the farming of Atlantic salmon and is seeking to double its economic contribution to £3.6 billion by 2030 [34]. In addition to providing colour and nutrition, carotenoids are powerful antioxidants and are used as preservation agents to slow down the oxidation process, therefore reducing food degradation and development of off-flavours [35].

In cosmetics, carotenoids are used as active ingredients with biological activity in creams and lotions. Similarly to food/feed, carotenoids are also used in cosmetic products for their antioxidant properties and nutritional value to the skin and hair. Carotenoids protect humans from UV-light damage and are applied externally via skin (as a topical treatment) as well as via dietary means [36]. Interestingly, they are also used in tan lotions because of their colour. The rising demand for natural, herbal and organic beauty products creates potential for carotenoids derived from microalgae (e.g., Eclae cosmetics [37]).

The health and nutraceutical market is currently the fastest growing sector of the carotenoids industry, projected to reach $440 million in 2019 with a CAGR of 3.7% [29]. Carotenoids are in rising demand as a source of dietary supplements. Free radicals, oxidative stress and cell redox imbalance have been identified as key culprits of a wide range of human diseases, including degenerative diseases [38]. As antioxidants, carotenoids neutralise free radicals and therefore prevent or slow down chronic diseases, cellular damage and aging [39]. There are many reviews highlighting the benefits of carotenoid to human health [40]. Specifically, it has been reported that carotenoids reduce the risk of inflammation, heart disease, and cancer [39], type 2 diabetes [41], chronic eye and macular diseases [42], obesity [43], Alzheimer’s disease, Parkinson’s disease, and amyotrophic lateral sclerosis (ALS) [38] and carotenoids could serve as therapeutics against other mental diseases as well [44]. With aging populations, people are becoming increasingly health conscious and aware regarding age-related problems and life-style-related diseases (Table 1).

## 6. Natural versus Synthetic Carotenoid

The amount of natural carotenoids produced and subsequently extracted from plants, animals, and micro-organisms is very small. Even the highest producing strains of microalgae synthesize <10% of carotenoids per DW. Carotenoids can be rapidly produced synthetically using low-cost labour and inexpensive chemicals, negating the need for the presence of a living organism and subsequent harvesting and extraction costs. Although synthetic carotenoids are faster and cheaper to produce, they are less effective in terms of their health-promoting properties and are hence less valuable and desirable as a product. For instance, the price of a microalgal-derived carotenoid can reach > $7,500/kg [45], whereas its synthetic counterpart may be half that cost [46].

Synthetic carotenoids are produced using a variety of methods, including reactions of dehydration and elimination, selective condensation of carbonyl compounds and homo-dimerization reaction, and selective coupling reaction of Csp^2^–Csp^2^. The commonly used Wittig reaction combines two phosphonium salt molecules (each C15) and one dialdehyde molecule (C10). The reaction product (C40) undergoes isomerization to form β,β-carotene, lycopene, or astaxanthin [47]. 

Microalgal β-carotene is a natural mixture of all-trans and 9-cis isomers with many health benefits. However, synthetic β-carotene is only composed of the all-trans isomer and does not possess the same beneficial properties. Similarly, natural microalgal astaxanthin is more than 95% esterified. This means that fatty acids are attached to one or both ends of the molecule. By contrast, synthetic astaxanthin is all free form, or unesterified. In comparison to its synthetic equivalent, naturally produced astaxanthin was found to be 50 times stronger in singlet oxygen quenching and 20 times stronger in the neutralization of free radicals [48]. The Natural Algae Astaxanthin Association (NAXA), based in Texas, is an association of manufacturers of natural astaxanthin derived from *Haematococcus pluvialis*. The NAXA lists natural astaxanthin manufacturers and products on their website and provides a NAXA Verification Seal on approved products. Currently, there are five manufactures on the NAXA list (AlgaTechnologies, Cyanotech, Atacama bio, Yunnan Alphy Biotech and AlgaeHealth) [49].

Given the high cost and low production of natural carotenoids, synthetic equivalents must be produced to satisfy market demands. As a result, synthetic carotenoids are ubiquitous, with a market share of 76% in 2013 [29] and are more widely accepted in developing regions such as Asia-Pacific, as the consumers find it convenient and affordable to purchase such products. However, the market for natural carotenoids is projected to grow. Consumers prefer to use natural products over synthetic owing to increasing health consciousness, awareness, and ill-effects of the synthetic products, especially in the European and the North American regions. 

The EU also has several standards restricting the use of harmful and toxic products/additives in food and feed. In 2017, the European Commission suspended the use of antioxidant ethoxyquin (EQ) as a feed additive [50]. The EU Commission concluded that there is insufficient evidence to prove that there is no genotoxicity and no adverse effect of EQ on animal health, human health or the environment. EQ was used as both stabilizer (to prevent feed degradation) and also to prevent the combustion of raw materials during transport and storage. This EQ case highlights the need for natural antioxidants like carotenoids and pressures manufactures into using natural antioxidant feedstocks at a large scale. 

## 7. Mass Production of Microalgae 

The mass production of microalgae is performed in enclosed photobioreactors (PBRs), open ponds or a combination of both. These systems have contrasting advantages and disadvantages. The main factors to consider when selecting a cultivation system are capital cost, operational cost, area available for cultivation, climate (light, temperature, rain, etc.), risk of contamination, water availability, mixing strategy, level of automation and system efficiency, and the purpose of cultivation [51].

In addition to selecting the most suitable cultivation system, phycologists also face a decision of growing a naturally occurring species (a wild type) or potentially genetically modified organisms (GMO) in which targeted properties (e.g., carotenoid production) may be enhanced. Farming GMOs is a controversial topic in many countries [52] and large commercial cultivations of GMOs are rare. When they do occur (e.g., Algenol in Florida, USA), they are always in enclosed PBRs and have strict regulations and monitoring and containment plans are installed to prevent GMOs from escaping. 

**Figure 2:**
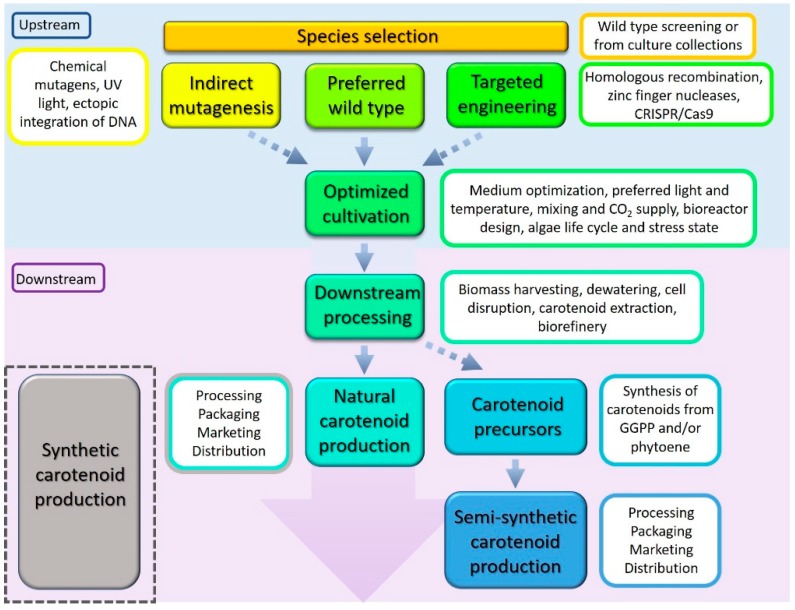
Pathways to market: Microalgae cultivation process for carotenoid production. Fully synthetic carotenoid production does not include any living organisms or upstream processing. The broken arrows denote alternative approaches to market.

Carotenoid production strategies using microalgae are highlighted in Figure 2. The first option is to screen microalgal genera to select a species/strain that naturally produces high levels of carotenoids. The second option (where legislature and consumer acceptance allows) is to select a suitable species and genetically engineer this species to increase the production of carotenoids. This genetic change can occur either indirectly via mutagenesis or directly via targeted engineering. For both options, the selected species is then cultivated in a way that further enhances carotenoid production. There are various biotic and abiotic conditions that can favour carotenoid production, including high light, osmotic shock, and nutrient stress, or any condition that pushes the cell into stationary phase of the growth cycle. Large-scale microalgal cultivation commonly follows a two-step procedure; firstly, cultivating the cells quickly to accumulate large volumes of biomass before applying a stressor to drive the cells to synthesise molecules of interest, e.g., carotenoids. 

## 8. Biomass Harvesting and Carotenoids Extraction 

Harvesting (removing microalgae from a cultivation medium) is not trivial due to the microscopic size of the cells. Even at their highest densities at the end of the growth cycle, microalgae constitute only a small fraction of the medium despite the deceiving rich colour (e.g., high pond density of 1 gram of dry algae per litre is only ~0.1% of weight). Dewatering techniques vary dramatically with resources available and range from settling, flocculation, filtering, centrifuging and a combination of these strategies [53]. Technologies are rapidly evolving and include advanced settling tanks, membrane filters, oscillating filters, dissolved air floatation systems, hydrocyclones, electrocoagulation, flow through centrifuges and even scalable fractionation [53,54].

Extraction and processing of carotenoid pigments from harvested microalgal biomass is also challenging. For example, *Nannochloropsis* has a very small surface area to volume ratio coupled with a multi-layered cell wall, making it extremely recalcitrant to disruption [55]. Cell lysis and carotenoid extraction can be achieved using several methods (and their combinations) including physical grinding, milling, ultrasound-assisted extraction, microwave-assisted extraction, freeze-thawing, supercritical fluid extraction, enzyme-assisted extraction and solvent extraction [56]. Carotenoid extraction using organic solvents (e.g., acetone, methanol, ethanol, hexane, dodecane) at a higher temperature and pressure are the most popular and are standardized to meet commercial specifications [27], whereas novel pre-treatment and extraction processes such as enzyme-assisted extractions, use of green solvents (environmentally safe and non-toxic solvents), subcritical water extraction and super-critical CO_2_ extraction (non-toxic, non-flammable method) are becoming increasingly prevalent [56,57]. For example, Gallego et al. [58] optimized the use of compressed fluid-based techniques for green extraction. The obtained products are routinely tested for quality and safety before being procured by food, feed, cosmetics, and supplement manufacturers. Another option is to avoid extraction of carotenoids and to use the whole microalgal biomass instead. In addition to carotenoids, microalgal cells can contain other beneficial compounds (e.g., omega 3 fatty acids, nutritious amino acids and carbohydrates, etc.). If the final application allows (e.g., animal feed), whole biomass can be used which would provide additional nutritional benefits even though carotenoid effect would be diluted by this extra biomass. 

There is a whole range of parameters to consider when selecting the appropriate harvesting, dewatering and extraction technologies to be employed. For instance, the capital and operational expenses, size of the algal enterprise, the degree of dehydration required, the final application of the biomass/extract, the required form of the collected biomass/extract and its residue. Up- and down-stream processing (including algal cultivation, carotenoid harvesting and extraction, product purification) is the most cost intensive part of the supply chain. Therefore, lowering the cost associated with processing is crucial for making the commercialization process more effective.

## 9. Legislations and Regulatory Aspects 

**Figure 3:**
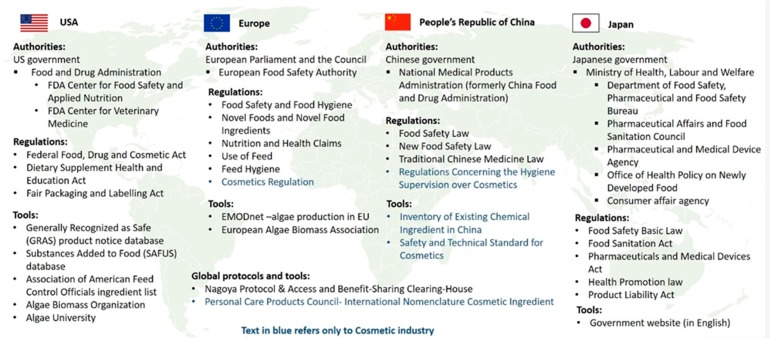
List of authorities, regulations and tools for each geographic region.

Safety and regulatory requirements vary among different countries and geographical areas [59]. Companies which intend to sell products containing microalgae (or their active compounds) must comply with the regulations in place which generally means to apply to a region-specific authority for approval, submit scientific information and health and safety assessments (Figure 3). 

Region-specific regulations are highlighted below and in Figure 3. There are global initiatives such as the United Nations’ Nagoya Protocol, that we have described in detail as well (Section 9.4). Several international tools and databases are also mentioned. For example, Personal Care Products Council (PCPC) publishes a worldwide science-based dictionary of labelling names for cosmetic ingredients called International Nomenclature Cosmetic Ingredient (INCI) [60]. The objective of INCI is to maintain a database of systematic names internationally recognized to identify cosmetic ingredients. PCPC also links to the International Regulatory Database (IRDB) which is database of regulations for cosmetics and personal care products in > 60 countries [61]. IRDB contains rules on basic health laws, cosmetic regulations, packaging and environmental rules, and regional industry guidelines. This information is available only to paying subscribers and was not examined by the authors of this manuscript. 

### 9.1. Europe

Europe is the largest market for carotenoids and it is projected to grow in next five years. In 2016, the carotenoid market value was $466 million [62]. This is a partly because Europe is a home base for leading carotenoids manufacturers (e.g., Germany, the Netherlands and Denmark) that export worldwide and because of changing societal awareness regarding health and diet. 

In 2017, the European Committee for Standardization (CEN) established a technical committee (TC 454) of > 30 experts to develop standardization deliverables for “Algae and Algae Products”. This mandate is for 5 years and the goal is to develop new European standards that will improve the reliability of the algae supply chain, increase consumer/industry trust of the algae market and enhance the algae market overall [63]. In 2014, prior to regulations update, the EU JRC Scientific and Policy Report showed that 33% of respondents identified “regulatory approval” as a key challenge to Europe’s competitiveness in the microalgae-based industry [64]. However, this issue was raised again in 2019 at a workshop on “Algae production in Europe” [65], where the consensus was that despite recent simplification of legislation, it remains problematic to meet existing regulations. 

The European Algae Biomass Association (EABA) is an association that promotes algae-related research, development of technology and industrial capacities, and mutual cooperation between scientists, industry and policy makers. The EABA is promoting algal-interest at both European and international levels [66]. Part of this effort is to compile a list of 30–40 microalgal strains that will be submitted to the EU as novel foods and/or ingredients with the hope to serve as a catalyst and expedite the approval process.

#### 9.1.1. European Regulations: Food/Feed and Nutraceuticals 

In Europe, three levels of regulations apply to the marketing of microalgae or its components: (1) general food safety regulations, (2) novel food ingredients, and (3) nutrition and health claims for food. 

(1) Market introduction of food products using the whole microalgal cells (e.g., *Chlorella*) or products that include the microalgae are subject to the food safety regulations that apply to all food products, such as the European Parliament and the Council’s regulation on *Food Safety* EC No 178/2002 [67] and regulation EC No 852/2004 regarding the hygiene of foodstuffs [68]. These regulations ensure a high-level protection of public health, establishing common principles and the basis for the assurance of human health protection. 

(2) The *Novel Foods and Novel Food Ingredients* regulation (EC 258/97) was replaced by EU 2015/2283 in 2018 in order to simplify the current authorisation procedures to take into account recent technological progress and to review, clarify and update the categories of food which constitute novel foods [69]. These foods cover whole insects and their parts, as well as food from cell culture or tissue culture derived from animals, plants, microorganisms, fungi or algae and food of material of mineral origin. EU 2015/2283 applies for foods and food ingredients that were not on the European market and consumed significantly before 15 May 1997. For example, *Spirulina* has been on the market prior to 1997, and is hence not categorized as novel food; however, the blue pigment, phycocyanin, that it produces is considered as a new product and thus falls under the Novel Food Law [64]. 

The Novel Food Law introduces more appropriate, less time-consuming and most importantly, centralized assessment procedures for food new to the EU. The European Food Safety Authority (EFSA) [70] conducts a scientific risk assessment regarding human health and safety. In parallel, the European Commission manages the files of each applicant and puts forward a proposal for the authorisation of a novel food which is found to be safe. Once the novel food is authorised, it can be consumed in any EU Member State. Newly developed scientific evidence and proprietary data will not be able to be used for the benefit of another application for 5 years after the novel food has been authorised [71].

(3) In 2006, the European Regulation on *Nutrition and Health Claims made on Foods* was introduced with Regulation EC No 1924/2006 [72]. This regulation states that health claims on food/feed products shall be based on and substantiated by generally accepted scientific evidence. There are two types of generic health claims: (A) nutrition claims, such as “source of” or “rich in” and (B) health claims implying that an ingredient has health benefits, such as "Magnesium helps reduce fatigue". Specific health claims can also be submitted with detailed scientific evidence including results of clinical studies. Health claims are authorized in the EU after a scientific assessment of the highest possible standards. In order to ensure harmonized scientific assessment of these claims, the European Food Safety Authority (EFSA) is carrying out such assessment. Labels cannot contain claims that their product will diagnose, cure, mitigate, treat or prevent a disease. Between 2008 and 2011, the EFSA evaluated 2758 food-related general health claims to see if they were supported by scientific evidence. Only around 10 percent of the claims could be substantiated [73].

The production of animal feed is an important component of EU agriculture and aquaculture. Feed may take the form of feed materials, compound feed, feed additives, premixtures or medicated feedingstuffs (this definition does not include water). There are two regulations in place: Regulation EC No 767/2009 on the placing on the market and use of feed [74] and Regulation EC No 183/2005 laying down requirements for feed hygiene [75]. The goal of both regulations is that all feed businesses, including aquaculture, operate in conformity with harmonised safety requirements.

#### 9.1.2. European Regulations: Cosmetics 

In Europe, Cosmetic Regulation 1223/2009 on cosmetic products is the main regulatory framework for finished cosmetic products when placed on the EU market [76]. It strengthens the safety of cosmetic products and streamlines the framework for all operators in the sector. This regulation replaces Directive 76/768/EC. 

EU Cosmetic regulation defines a cosmetic product as “any substance or mixture intended to be placed in contact with the external parts of the human body (epidermis, hair system, nails, lips, and external genital organs) or with the teeth and the mucous membranes of the oral cavity with a view exclusively or mainly to cleaning them, changing their appearance, protecting them, keeping them in good condition or correcting body odours” [77].

The choice of ingredients contained in cosmetic products must comply with Article 14 of Cosmetic Regulation, specifically Annexes II–VI which list prohibited substances, list substances authorized under certain conditions, a positive list of dyestuffs, a positive list of preservatives, and a positive list of sunscreens, respectively. 

Cosmetic regulation 1223/2009 introduces the notion of a *Responsible Person*. This person must take responsibility to ensure that every cosmetic product placed on the EU market complies with all the requirements of the Cosmetic Regulation. If there are any concerns about its safety, its packaging or its labelling, the responsible person will be considered liable and can be penalized. 

A Responsible Person must hold specific product and safety information accessible to control authorities when requested. Product Information Files (PIF) are maintained under the EU Cosmetics Regulation. The PIF must include the product description, the Cosmetic Product Safety Report (CPSR), details of methods of manufacture in accordance with cosmetic Good Manufacturing Practice (cGMP) and, where justified, proof of the effect claimed. The selection of the cGMP is, in principle, voluntary, but the Cosmetics Regulation provides an incentive to follow the European Standard ISO 22716:2007 [78]. If a product follows this standard, which is recognized as a harmonized standard by the EU, it benefits from a presumption of compliance with the Cosmetics Regulation’s cGMP requirement.

A significant change to Cosmetic Regulation is that the Responsible Person is required to notify Serious Undesirable Effects (SUE) to the authorities and that colourants, preservatives and UV-filters, including those that are nanomaterials, must be explicitly authorised. Manufacturers need to notify their products via the EU Cosmetic Products Notification Portal [79]. The European Commission database for information on Cosmetic Substances and Ingredients (COSING) is a searchable database that lists all active cosmetic ingredients. Currently, COSING database returns 30 active ingredients under the search “*Chlorella*” [80]. 

### 9.2. United States of America

North America is the second-largest market for carotenoids in the world (after Europe). In 2016, the market value reached $371 million [62], which was higher than the 2019 projection of $355 million, suggesting that CAGR of 3.5% was an under-estimate [29]. 

The US congress encouraged algal cultivation efforts by including algae as an agricultural commodity in the 2018 Farm Bill. This means that the US Department of Agriculture (USDA) now supports algae as a crop in a variety of ways; from research and market programs to crop insurance [81]. Algae are also specifically named as a pathway for carbon capture qualifying for so-called 45Q tax credit. New legislation called “Utilizing Significant Emissions with Innovative Technologies (USE IT) Act” has also been proposed [82]. This bill would support new technologies that capture carbon from industrial sources. While these initiatives are very beneficial to large-scale algae cultivation, the algae industry is still relatively new and faces many challenges. US-based companies focusing on algae production are mapped out on the Algae University website [83].

#### 9.2.1. US Regulations: Food/feed and Nutraceuticals 

In the US, the Food and Drug Administration (FDA) Center for Food Safety and Applied Nutrition (CFSAN) is responsible for regulating food ingredients and ensuring that those ingredients are safe and lawful. 

The FDA is authorized by the US government to regulate both laws that are applicable to microalgae-based food and feed products available on the consumer market: Federal Food, Drug and Cosmetic Act (FD&C Act Title 21 of the United States Code) introduced in 1938, which regulates all foods and food additives [84] and the Dietary Supplement Health and Education Act (DSHEA) introduced in 1994, which amended the FDC Act to cover dietary ingredients and supplements. A dietary ingredient is defined as “*a vitamin; mineral; herb or other botanical; amino acid; dietary substances for use by man to supplement the diet by increasing the total dietary intake; or concentrate, metabolite, constituent, extract or combination of the preceding substances*”. A dietary ingredient which was not marketed in the US before 15 October 15 1994 is considered a new dietary ingredient (NDI). In that case, a NDI pre-notification must be made to the FDA at least 75-days before the ingredient is on the market.

The FDA also regulates and assigns a Generally Recognized as Safe (GRAS) status to a product. The GRAS process is specific to the conventional food and food additives. GRAS notices are publicly available in the FDA inventory [85]. A GRAS notice is typically > 100 pages long and covers intended uses and concentration, experimental results, literature searches and more. For example, GRAS no 754 is Corbion’s request for use of algal oil (87% oleic acid) derived from alga *Prototheca moriformis* and it was approved as *safe* in July 2018 [85]. A searchable database of approved Substances Added to Food US (SAFUS, formerly EAFUS) is also available for public use [86]. The GRAS self-affirmation requires a complete review and assessment of the safety of the ingredient. Results of toxicology studies must be previously published. The process of achieving GRAS self-affirmation requires a consensus of safety by a panel of experts who are qualified by training and expertise to evaluate the safety of food and additives. They then obtain the GRAS status; the FDA must review the GRAS notice and answer with a “no question” letter. 

The authorization of feed products falls under the FDA Center for Veterinary Medicine (CVM). The Association of American Feed Control Officials (AAFCO) annually publishes feed ingredient definitions in the AAFCO official publication [87]. 

The FDA does not regulate the use of the term “organic” on food labels. The National Organic Program (NOP) is the federal regulatory framework regulating organically produced crops. USDA oversees the program and enforces the NOP regulations and standards. All labelling required by the regulations must be in English, except for products distributed solely in Puerto Rico or a territory where the predominant language is not English. 

#### 9.2.2. US Regulations: Cosmetics 

In the US, the two most important laws pertaining to cosmetics market are the Federal Food, Drug, and Cosmetic Act [84] enforced by the FDA and the Fair Packaging and Labelling Act [88] governed by the Fair Trade Commission.

The purpose of these acts is to ensure that food, drugs, medical devices, and cosmetics are safe to use, properly labelled and to prevent unfair or deceptive packaging and labelling. The following information must be displayed on the principal display panel (FDA Code of Federal Regulations Title 21 Part 701 Cosmetic Labelling): an identity statement indicating the nature and use of the product, an accurate statement of the net quantity of contents, name and place of business or distributor statement, material facts, warning and caution statements, ingredients listed in descending order of dominance.

### 9.3. Asia

Asian countries have enjoyed benefits of algae for centuries. Customers are more aware of the potential of microalgae due to this rich cultural history when compared to the West. The labour cost of cultivation is generally cheaper and less regulated [89]. Both China and Japan are countries interested in the microalgal-based industry because of the desire to open sustainable new markets in food, feed, nutraceuticals and cosmetics. Japan is also keen to tackle its vulnerable energy supply situation by investigating alternative energy sources, including algae-based biofuels. In 2016, 94% of energy consumed was imported [90]. Furthermore, In Japan, research activities and mass cultivation of *Spirulina* for use as a foodstuff began in the 1970s [91]. There are several active microalgae cultivation companies in China (e.g., Beijing Ginko Group and AlgaeHealth), Japan (e.g., Euglena and DENSO Corporation), Taiwan, Thailand and India that are growing in size but many more projects are in the R&D phase [92]. 

#### 9.3.1. Regulations in the People’s Republic of China: Food/feed and Nutraceuticals 

The Chinese government regulates food production and processing as well as food packaging, additives, drug production, and trade regulation [93]. The Food Safety Law, which was amended in 2015, is enforced by the National Medical Products Administration (NMPA) (formerly the China Food and Drug Administration). The New Food Safety Law covers new raw materials and their function. A history of food safety scandals (e.g., melamine-tainted milk powder) prompted the formation of new guidelines called “Opinions on Deepening Reform and Strengthening Food Safety Work” published in 2019. These guidelines propose stricter standards, supervision, sanctions and accountability [94]. 

Health foods are regulated under the Food Safety Law and are being increasingly recognized. The legal status of health food was first recognized in the law in 1996 and since then, there have been 16,494 approved health foods in China in 2017 and 785 approved imported health foods [95]. The first law on Traditional Chinese Herbal Medicine came into force in 2017 covering standardization to ensure quality and safety of herbs in cultivation, collection, storage and processing [96]. Seaweed, microalgae, and cyanobacteria are used as health foods and nutraceuticals and marketed products range from raw seaweed to *Spirulina* tablets. 

#### 9.3.2. Regulations in the People’s Republic of China: Cosmetics 

In China, the regulation of cosmetics falls under Regulations Concerning the Hygiene Supervision over Cosmetics. It defines the substances that may or may not be present in a cosmetic product. The verification of the formula must be done according to two reference systems enforced by the National Medical Products Administration (NMPA): 1) Inventory of Existing Chemical Ingredient in China (IECIC), a list including all the ingredients known and allowed in cosmetic products on the Chinese market (8783 ingredients in 2014 [97]) and 2) Safety and Technical Standard for Cosmetics (STSC), the Chinese cosmetics regulation which presents several lists of substances, with a similar wording to the European cosmetics regulation equivalent. It consists of general, forbidden and restricted ingredients, positive lists of preservatives, colorants, hair dyes, and UV filters. In addition, it lists methods used to test the physico-chemical properties of an ingredient, microbiological analyses, toxicology, human security, and evaluating efficacy [98]. 

#### 9.3.3. Regulations in Japan: Food/feed and Nutraceuticals

In Japan, food safety is governed by the Minister of Health, Labour and Welfare (MHLW) as part of the Department of Food Safety, under the Pharmaceutical and Food Safety Bureau. There is also an Office of Health Policy on Newly Developed Food. The main law that governs food quality and integrity is the Food Safety Basic Law (FSBL) and related laws such as the Food Sanitation Law (FSL) [99]. The Food Safety Basic Law was established in 2003 after several public concerns, including the occurrence of bovine spongiform encephalopathy (BSE, commonly known as mad cow disease) in 2001. FSBL is responsible for risk assessment while FSL is responsible for risk management. The FSL has various responsibilities, including food standardization, food additives, packaging, hygiene management, quality assurance and business licenses [99]. The MHLW ultimately declares products safe after consultation with the Pharmaceutical Affairs and Food Sanitation Council (PAFSC). In addition, it is not permissible to add vitamins, minerals, novel foods or nutritive substances to food unless they have been expressly declared by the MHLW as having no risk to human health.

Foods with health claims are either classified as Foods with Nutrient Function Claims (FNFC) or Foods for Specified Health Uses (FOSHU). The Food Safety Basic Law, the Food Sanitation Law, the Health Promotion Law (Act No. 103 of 2002, amended in 2018) and the Pharmaceuticals and Medical Devices Act apply for both FNFC and FOSHU (Figure 3) [100,101]. The FNFC is labelled with the nutritional claims specified by the MHLW. Specifications for 17 ingredients (12 vitamins and 5 minerals) have been established and foods containing these ingredients can be manufactured and sold without permission from MHLW provided that strict standards are met. FOSHU are foods that contain ingredients with beneficial physiological functions and biological activity. These foods require a detailed review process with scientific evidence for each application. Each FOSHU application must include three sets of documents: (1) clinical studies demonstrating the effectiveness of FOSHU (2) safety documentation and (3) analytical determination of the functional component. MHLW in close collaboration with the Council on Pharmaceutical Affairs and Food Safety Commission must evaluate each application within one year. Labels of both FNFC and FOHCU must contain a detailed nutrient content, functionality claims, recommended dosage and administration, method of consumption and any warnings if applicable [100]. 

#### 9.3.4. Regulations in Japan: Cosmetics 

Similarly to the food/feed and nutraceuticals regulation, the Japanese government regulates the cosmetics industry through its Ministry of Health, Labour and Welfare (MHLW) in conjunction with the Pharmaceutical and Medical Device Agency. The Pharmaceuticals and Medical Devices Act (PMDA) established in 2014 replaced the pre-existing Pharmaceutical Affairs Law. The PMDA consists of 17 chapters and 91 articles, including quasi-drugs and cosmetics (Articles 12 to 23). The current PMDA regulations covers securing quality, efficacy and safety of pharmaceuticals, medical devices, regenerative and cellular therapy products, gene therapy products, and cosmetics [102].

The safety of cosmetics in Japan is ensured by the Product Liability Act (Law 85 of 1994). The burden of ensuring product safety has been shifted to cosmetic manufacturers; however, the Japanese government maintains a list of approved active ingredients and UV filters, lists of prohibited ingredients and of restricted ingredients [103].

### 9.4. The Nagoya Protocol 

The Nagoya Protocol on Access to Genetic Resources and the Fair and Equitable Sharing of Benefits Arising from their Utilization to the Convention on Biological Diversity (CBD) was adopted in Japan in 2010 and came into force in 2014. The Nagoya Protocol is a supplementary agreement under the UN Convention on Biological Diversity and its objective is the fair and equitable sharing of benefits arising from the utilization of genetic resources. Under the Nagoya Protocol, each country has the right to regulate access and utilization of their genetic resources and associated traditional knowledge. Countries can also negotiate any benefits resulting from sharing these genetic resources. The main goal of the Nagoya protocol is to contribute to the conservation, sustainable use of biodiversity and fair sharing of genetic resources. The protocol has 92 signatories and 120 parties [104]. Parties are urged to publish all mandatory information available at the national level onto the Access and Benefit-Sharing Clearing-House (ABSCH) searchable database [105].

Under the Nagoya Protocol, microalgal culture collection facilities have a responsibility to demonstrate due diligence during material isolation, storage and distribution. This has been improved by electronic visibility through links to global biodiversity and genomic databases [106]. For example, the Culture Collection of Algae and Protozoa (based in the UK) is currently using governmental guidance for advising, supporting and enforcing the EU Access and Benefit Sharing regulation [107,108]. However, since the US did not ratify the Nagoya Protocol, US Culture collections officially follow policies of the USA and other countries regarding access to genetic resources [109].

## 10. Opportunities and Challenges 

**Figure 4:**
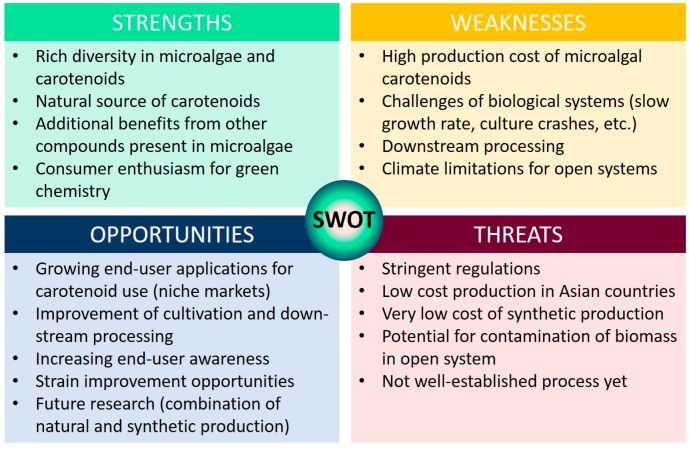
SWOT analysis of production of carotenoids from microalgal sources.

In this section, the strengths, weaknesses, opportunities and threats (SWOT analysis) of microalgae-based carotenoid markets are discussed (Figure 4). This provides an appraisal of the current perspective and outlook of the global algal carotenoid markets. 

### 10.1. Strengths and Opportunities 

Carotenoids and their antioxidant properties continue to generate significant interest in multiple markets, including food and feed, nutraceuticals, and cosmetics. The growth of the aquaculture industry, awareness about the medicinal applications, and increasing consumer preference for natural products, carotenoids from microalgae are well positioned for growth in revenue [27]. There is also a large market opportunity in the development of optimized microalgal cultivation systems (including intelligent screening of microalgae and strain improvement), more efficient and environmentally friendly carotenoid extraction methods, and downstream processing techniques with an emphasis on the circular economy and sustainability. The end-user is also ever-evolving with increasing interest in Lifestyles of Health and Sustainability (LOHAS) which prioritize the production of natural carotenoids from sustainable sources.

There are many opportunities for future research, including emerging carotenoids, novel applications and technologies. The development of markets (both niche and commodity) will expedite the research as well. 

### 10.2. Weaknesses and Threats 

Natural carotenoid-rich microalgae are challenging to produce as it can be a labour intensive and an expensive process. Carotenoid-producing microorganisms are generally characterized as having a low growth-rate and target carotenoid productivities, coupled with being recalcitrant, making the extraction of the carotenoid even more problematic. Low-cost competition (involving lower labour and production costs in some Asian countries such as China, Japan and India) may appear as market restraints to the western carotenoid markets. However, these Asian markets provide affordable carotenoids to a high number of consumers. 

Given the chirality in isomers, it is difficult to analytically distinguish between natural and synthetic carotenoids in a laboratory. Only specialised techniques such as isotope ratio mass spectroscopy can be reliably used in authenticity assessments [110]; hence, it is very easy for the adulterated version (synthetic pigments) to gain entry in the market, falsely claiming that they are natural products. Due to this, adulterated products are being sold in the market to gain profit. Lastly, strict regulations and slow-moving legislations may hinder the route to market and development of innovative products.

A recent report on algae production in Europe (Report on the Community of Practice Workshop: Algae production in Europe: status, challenges and future developments, see [65]) found that despite the growing interest in algal resources, production in Europe lags behind the production at the global level. This is driven by the scale and competitiveness of Asian algal aquaculture and the scarcity, ambiguity and fragmented information available on the sector. This report also concluded that existing regulatory framework for the algae sector might need to be optimised to take into account market data and to develop a database on algal production and targeted markets [65]. 

Similarly, a recent opinion paper [111] mentioned that biodiesel production from microalgae was also hyped in early 2000, yet only few developments made it to the market. However, within the same commentary, the authors highlighted that microalgae are suitable for the production of high-value products, especially food/feed and nutraceuticals, but still face some challenges for their widespread applicability [111].

## 11. Major Players, Current Trends and Future Research 

Currently, the German company BASF SE, which is the largest chemical producer in the world leads the production of synthetic carotenoids under the brand names Lucantin ® and Lucarotin ® [112]. Second to this is DSM, a Dutch company that produces synthetic carotenoids under the brand name Carophyll® [113]. Other smaller companies (e.g., Döhler Group (Germany), Chr. Hansen (Denmark) and Kemin Industries (USA)) are also well-established synthetic carotenoid producers that have responded to a rising demand and pressure for natural carotenoids. As a result, these companies expanded their portfolio to include natural carotenoids. For example, DSM in partnership with Kemin introduced natural zeaxanthin into its product Optisharp®, in 2014. Kemin (USA) produces a line of natural and organic carotenoids sourced from paprika, kelp and marigold [114]. Vitatene (Spain), also partnered with DSM, is a company that is sourcing carotenoids from a fungus (*Blakeslea trispora*) [115]. 

Many microalgal producers in the western hemisphere (e.g., Algalif, Fermentalg, Allmicroalgae Natural Products, BDI-BioLife Science, Qualitas Health, Earthrise, Triton, A4F) are already involved in carotenoid production because of the potentially high monetary rewards and current economic momentum. The microalgae-based carotenoid industry is dominated by Cyanotech (founded in 1983) which has been producing astaxanthin (and cultivating *Haematococcus* and *Spirulina*) for decades. The European Marine Observation and Data Network (EMODnet) recently added “algae production” to their human activities’ portal (Figure 3). Algal production facilities can now be viewed on the map and data can be downloaded (“EMODnet portal,” 2018). This portal can provide tips on what algal products are already on the European market and possibly assist with species selection.

Strategic partnerships, collaborations and multi-disciplinary teams are key for surviving competitive markets and there seems to be a recent trend towards this with relation to the algal carotenoids. For example, AlgaTechnologies Ltd (Israel) has recently teamed up with Sphera (Italy) allowing AlgaTechnologies to cultivate algae and produce astaxanthin and fucoxanthin and Sphera to encapsulate the product [116], whereas the Chinese astaxanthin producer Beijing Ginko Group (BGG), which is known for its algal production facility in Yunnan province (BGG subsidiary AlgaeHealth), has joined Natural Astaxanthin Association (NAXA) and announced the production of the “world’s first organic astaxanthin crop” in 2016 [117]. Additionally, BGG has joined forces with Solix (USA) to establish a new company based in Colorado focusing on the natural extraction of ingredients from algae and other feedstocks [118]. Lastly, the Israeli company Algaennovation brokered a deal with an Icelandic geothermal plant to receive environmentally friendly electricity, hot and cold water and carbon dioxide energy for their microalgae farm in Iceland [119]. 

Currently, only a fraction of carotenoids is being commercially utilized. There are many opportunities for future research, including further explorations of carotenoids and their applications, the development of high-value niche markets, and research of rare carotenoids, including those recently isolated from a handful of organisms. For example, the carotenoid myxol, can be found in freshwater cyanobacteria *Oscillatoria limosa*, *Anabaena variabilis* and *Nostoc commune* and has already been demonstrated to have stronger antioxidant activities when compared to β-carotene [59]. Other rare or recently described natural carotenoids include bacterioruberin, salinixanthin, saproxanthin, siphonaxanthin, and apocarotenoids, which are produced by bacteria, archaea, and algae [59,120]. Ongoing research focuses not only on novel carotenoids but also on innovative delivery methods. For example the European Union’s Seventh Framework Programme is supporting a project focused on bacteria-derived carotenoids that are gastric-stable and more bioavailable than common dietary carotenoids [121]. 

An unexplored area is also the use of natural precursors. Precursors, such as phytoene, are used independently and demonstrate many of the same qualities as carotenoids [28]. Since all carotenoids have a common precursor (Figure 1), there is a possibility of producing these naturally (e.g., GGPP or phytoene) within in a living organism but finishing the carotenoid production synthetically. Questions remain as to whether these “semi-natural carotenoids” can be easily produced, if they retain the qualities of fully naturally synthesised equivalents, and how expensive this is. Further research is necessary to validate this concept. 

## 12. Conclusions

The microalgal carotenoid market will most likely continue to expand due to increasing demand for natural products, the identification of novel carotenoids, advances in upstream and downstream methodologies, and growing market opportunities. Currently, there are existing opportunities for microalgal-based carotenoids in food, feed, nutraceuticals, and cosmetic industries. Legal aspects and regulatory process vary from country to country and are generally slow in catching up to scientific advancements. National authorities require scientific information and health and safety assessment before “approval” is issued. This process is slow and even more complicated if microalgal products are categorized as novel in which case no precedent exists. This review summarizes relevant authorities and regulations in Europe, USA, People’s Republic of China, and Japan and provides quick and broad introduction into the regulatory processes.

## Figures and Tables

**Table 1:** The role of carotenoids in microalgal cell and their benefits to human health.

Role of Carotenoids within Microalgal Cell		Benefits of Carotenoids to Human Health	
Membrane stabilization Light harvest Energy dissipation Active antioxidants Scavengers of reactive oxygen species	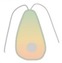	Lower risk of inflammation Lower risk of heart disease Cancer prevention Improved eye-health Protection of neurons Lower risk of type 2 diabetes